# Ultrahigh, Ultrafast, and Self‐Powered Visible‐Near‐Infrared Optical Position‐Sensitive Detector Based on a CVD‐Prepared Vertically Standing Few‐Layer MoS_2_/Si Heterojunction

**DOI:** 10.1002/advs.201700502

**Published:** 2017-12-01

**Authors:** Ridong Cong, Shuang Qiao, Jihong Liu, Jiansong Mi, Wei Yu, Baolai Liang, Guangsheng Fu, Caofeng Pan, Shufang Wang

**Affiliations:** ^1^ Hebei Key Laboratory of Optic‐Electronic Information and Materials College of Physics Science and Technology Hebei University Baoding 071002 P. R. China; ^2^ Beijing Institute of Nanoenergy and Nanosystems Chinese Academy of Sciences Beijing 100083 China; ^3^ CAS Center for Excellence in Nanoscience National Center for Nanoscience and Technology (NCNST) Beijing 100190 P. R. China

**Keywords:** heterojunctions, lateral photovoltaic effects, MoS_2_, position sensitive detectors, vertically layered structures

## Abstract

MoS_2_, as a typical transition metal dichalcogenide, has attracted great interest because of its distinctive electronic, optical, and catalytic properties. However, its advantages of strong light absorption and fast intralayer mobility cannot be well developed in the usual reported monolayer/few‐layer structures, which make the performances of MoS_2_‐based devices undesirable. Here, large‐area, high‐quality, and vertically oriented few‐layer MoS_2_ (V‐MoS_2_) nanosheets are prepared by chemical vapor deposition and successfully transferred onto an Si substrate to form the V‐MoS_2_/Si heterojunction. Because of the strong light absorption and the fast carrier transport speed of the V‐MoS_2_ nanosheets, as well as the strong built‐in electric field at the interface of V‐MoS_2_ and Si, lateral photovoltaic effect (LPE) measurements suggest that the V‐MoS_2_/Si heterojunction is a self‐powered, high‐performance position sensitive detector (PSD). The PSD demonstrates ultrahigh position sensitivity over a wide spectrum, ranging from 350 to 1100 nm, with position sensitivity up to 401.1 mV mm^−1^, and shows an ultrafast response speed of 16 ns with excellent stability and reproducibility. Moreover, considering the special carrier transport process in LPE, for the first time, the intralayer and the interlayer transport times in V‐MoS_2_ are obtained experimentally as 5 and 11 ns, respectively.

## 1 Introduction

Since the discovery of graphene in 2004, 2D materials have attracted significant interest due to their unique electronic and optical properties and numerous potential applications in optoelectronic devices.^1^, ^2^, ^3^, ^4^ Among various 2D materials, MoS_2_ has shown excellent properties in optoelectronic applications due to its suitable band gap value,^5^, ^6^ relatively high carrier mobility,^2^, ^7^ high light absorbance,^3^, ^8^ and, more importantly, good stability,^9^, ^10^ and brilliant optoelectronic properties.^1^, ^7^, ^11^, ^12^ However, pure MoS_2_‐based optoelectronic devices are usually limited to infrared light detection and lower photoelectric conversion efficiency (PCE) because of the direct band gap of 1.8 eV for single‐layered MoS_2_ sheet^5^, ^6^ and the picosecond ultrashort carrier lifetime.^13^, ^14^ To conquer the drawbacks of wavelength and lifetime limitations, van der Waals heterostructures,^15^ or lateral heterostructures,^16^, ^17^ which are made by stacking a monolayer on the top of another monolayer or a few‐layer crystal or controlled by epitaxial growth of lateral heterojunction, are developed and show great potential for designing high‐performance 2D material‐based photodetectors owing to the combined advantages and synergetic effects of different 2D materials with various band gaps and work functions,^18^, ^19^, ^20^ and the ultrafast layer‐to‐layer transfer speed of carriers.^21^ To date, various van der Waals heterostructures and lateral heterostructures have been prepared and successfully applied in photodetectors,^17^, ^22^ field‐effect transistors,^23^ photocatalysts,^24^ and solar cells.^16^, ^25^ However, large‐area defect‐free monolayer MoS_2_ has not yet been widely demonstrated and accurate transfer and positioning cannot be well achieved, making it difficult to utilize for large‐scale and high‐quality device applications as surface effects, such as surface band bending, surface roughness, photodesorption, gas chemisorption, and surface related defects or states, have significantly important effects on the photoelectric properties of 2D materials.^26^ Additionally, monolayer or few‐layer MoS_2_ can only absorb a small portion of incident light due to its ultrathin thickness, and its excellent optical absorption property cannot be well developed, thus restricting photocurrent (/photovoltage) improvement in MoS_2_‐based devices. Therefore, recently, researchers have tried to prepare large‐scale uniform multilayer^27^ or bulk MoS_2_ 28 and have conceived a feasible and easy way to construct high‐performance optoelectronic devices by combining a 2D layered material with a traditional Si semiconductor,^29^, ^30^, ^31^, ^32^ as Si still dominates the commercial photovoltaic or optoelectronic market due to high abundance, acceptable power conversion efficiencies, and mature semiconductor technologies. Although the optical absorption can be improved greatly, the existing numerous defects result in serious recombination, and good PCEs still cannot be obtained.^31^, ^32^ In addition, for 2D layered materials, the intralayer carrier mobility is supposed to be much higher than the interlayer carrier mobility; thus, one can imagine that the PCE or photoelectric response would still be undesirable, even for high‐quality parallel‐oriented multilayer or bulk MoS_2_‐based heterostructures. However, vertically oriented multilayer MoS_2_, which has the advantages of both strong light absorption and quick longitudinal in‐plane transport, may show huge potential in optoelectronic devices; e.g., in 2015, Wang et al. first deposited the vertically standing layered MoS_2_ directly on Si substrate to form MoS_2_/Si heterojunction photodetector by magnetron sputtering and realized an extremely high detectivity (≈10^13^ Jones) and an ultrafast response time (≈3 µs).^33^ Considering the outstanding properties and great potential in optoelectronic devices, recently, researchers have begun trying to synthesize other 2D materials of this vertically layered structure, and several large‐area, high‐quality, and vertically oriented few‐layered 2D nanosheets have been successfully prepared by chemical vapor deposition (CVD).^34^, ^35^, ^36^, ^37^ Notably, compared with other preparation methods, the quality of CVD‐prepared MoS_2_ should be much better due to the relatively intact layered structure and fewer defects. However, up to now, this research has mainly focused on single materials, and their heterojunctions have not been well fabricated and studied, which may be due to the difficulty of transferring the sheets or combining them with other 2D materials such as flatly stacked van der Waals heterostructures; thus, their photoelectric and time responses are still unknown.

In this paper, the large‐area, high‐quality, and vertically oriented few‐layered MoS_2_ nanosheets (V‐MoS_2_) were synthesized by CVD. Raman, scanning electron microscopy (SEM), and high‐resolution transmission electron microscopy (HRTEM) measurements suggest that the crystal quality of MoS_2_ nanosheets is very high with a layer thickness of approximately six layers. Moreover, the MoS_2_ nanosheets were transferred onto the p‐type Si substrate successfully, and then Au point electrodes were evaporated on the surface through a contact mask. Lateral photovoltaic effect (LPE) and optoelectronic effect measurements demonstrate that the V‐MoS_2_/Si heterojunction exhibits an excellent lateral photovoltaic performance in the wide range of visible‐near‐infrared light with ultrahigh sensitivity of 401.1 mV mm^−1^ and very good linearity (nonlinearity <5%) without applying an external bias voltage, giving it great potential for application in a self‐powered position sensitive detector (PSD). In addition, time‐response measurements suggest that this PSD has an unprecedented response speed, with a typical rise (/fall) time of 16 (/176) ns. More importantly, both the lateral photovoltaic and the time responses in the V‐MoS_2_/Si heterojunction are extremely stable and reproducible. Furthermore, the intralayer and the interlayer carrier transport times are obtained experimentally, for the first time, as 5 and 11 ns, respectively, by combining time‐response measurements of the V‐MoS_2_/Si heterojunction as a PSD and a photodetector. These results indicate that the CVD‐prepared V‐MoS_2_/Si heterojunction may have great potential application in high‐performance self‐powered photodetectors.

## 2 Experimental and Characterization

The vertically oriented layered MoS_2_ nanosheets were grown by CVD with appropriate control of the preparation parameters. Like most previously reported methods for growing transition metal dichalcogenides (TMDs), an alumina boat with ≈20 mg MoO_3_ powder and the growth substrate was loaded into the center of furnace heating zone 2, while another boat with ≈500 mg sulfur pellets was loaded into the center of upstream furnace heating zone 1. First, the sulfur and the MoO_3_ heating zones were heated to 65 and 160 °C, respectively, and the temperatures were maintained for 40 and 30 min, respectively, to remove the vapor from the raw materials and system. Then, the heating zones were heated to 150 and 760 °C for MoS_2_ preparation. During the process, Ar was introduced into the quartz tube as a carrier gas with a flow rate of 35 sccm under the atmospheric pressure. The schematic diagram of MoS_2_ growth and the temperature profile can be found in Figure S1a,b (Supporting Information).

To understand the entire growth process, the growth time was precisely controlled and set at 2, 4, 6, 10, 15, and 20 min based on the typical growth features. An ultrathin polygon flat layer, instead of vertically oriented MoS_2_ nanosheets, was observed at the beginning of growth, and the film exhibits two characteristic MoS_2_ Raman peaks ($E_{2g}^{1}$ mode at 385.9 cm^−1^ and $A_{1g}^{}$ mode at 405.7 cm^−1^) with a peak frequency difference of 19.8 cm^−1^ (as shown in Figure S2a, Supporting Information), which suggests that the MoS_2_ film is nearly a monolayer with a very uniform layer thickness.^4^, ^38^ With increasing growth time, the area increases and the MoS_2_ nanosheets become thicker gradually. After ≈4 min (Figure S2b, Supporting Information), vertically oriented nanosheets start to appear on the flat nanosheet, along with the enhancement of both the $E_{2g}^{1}$ and the $A_{1g}^{}$ peaks (relative to Si), and the peak frequency difference increases slightly (20.5 cm^−1^). When the growth time reaches 6 min (Figure S2c, Supporting Information), the density of the MoS_2_ nanosheets increases prominently in the in‐plane direction, and some nanosheets become hexagonally shaped. Then, with extended growth time (Figure S2d,e, Supporting Information), the MoS_2_ nanosheets become increasingly large and slightly thicker in both the in‐plane and out‐of‐plane directions with nearly equilibrium growth rate. Meanwhile, the intensities of both the in‐plane $E_{2g}^{1}$ vibration peak and the out‐of‐plane $A_{1g}^{}$ vibration peak are greatly enhanced (relative to Si). After ≈20 min, the uniform large‐area vertically oriented few‐layer MoS_2_ nanosheets (V‐MoS_2_) are obtained (Figure S2f, Supporting Information). As the Raman frequencies for the $E_{2g}^{1}$ and $A_{1g}^{}$ modes show opposing shifts with increasing layer thickness, the Raman peak frequency difference reaches 25.1 cm^−1^, and the Raman mapping measurement indicates that the MoS_2_ nanosheets exhibit the same peak intensity, suggesting that the vertically oriented nanosheets are of uniform thickness with only ≈6–7 MoS_2_ layers^38^ (as shown in Figure S3, Supporting Information).

**Figure 1:**
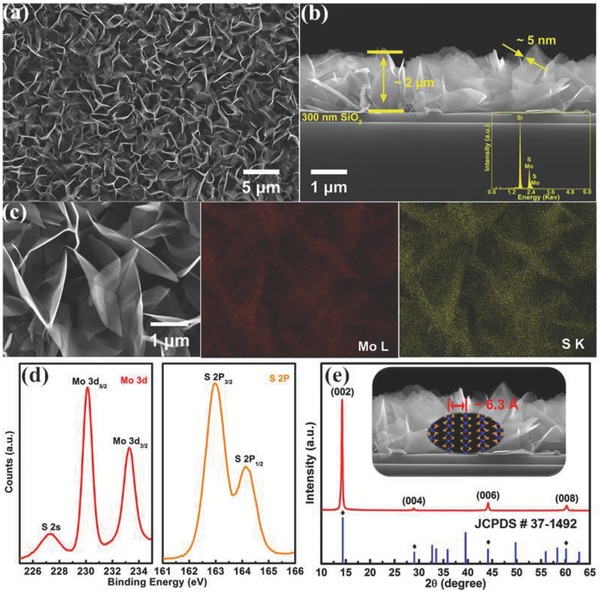
a) Top‐down, and b) cross‐sectional SEM images of V‐MoS_2_ (with the EDXS spectrum in the bottom‐right corner), c) SEM image of selected region (left) for EDXS maps of Mo (middle) and S (right) distributions, d) XPS spectra of Mo 3d and S 2p, and e) XRD result of V‐MoS_2_ nanosheets.

**Figure** **1**a presents the large‐sized SEM results of the MoS_2_ nanosheets, and the cross‐section image suggests that the height is ≈2 µm with bright and nearly transparent vertically oriented structure (Figure 1b), suggesting that the V‐MoS_2_ nanosheets are very thin (≈5 nm), which is consistent with our Raman results. Moreover, energy dispersive X‐ray spectroscopy (EDXS) compositional mappings of Mo and S distributions within MoS_2_ nanosheets are shown in Figure 1c. Referencing the SEM image of MoS_2_ nanosheets (the left side of Figure 1c), the distributions of Mo and S elements are essentially homogeneous, and the atomic ratio of Mo/S is ≈1:1.944, which is slightly substoichiometric. Chemical state analyses of the synthesized MoS_2_ nanosheets are also conducted using X‐ray photoelectron spectroscopy (XPS). As shown in Figure 1d, peaks at 162.98, 164.15, 230.10, and 233.29 eV are observed, corresponding to S 2p_3/2_, S 2p_1/2_, Mo 3d_5/2_, and Mo 3d_3/2_, respectively.^39^ Finally, the X‐ray diffraction (XRD) was measured to identify the crystalline structure of V‐MoS_2_ nanosheets (shown in Figure 1e). In addition to the sharp (002) prominent diffraction peak, higher‐order peaks of (004), (006), and (008) were observed, indicating long‐range crystalline order in the MoS_2_ nanosheets. It is evident from these results that the as‐prepared V‐MoS_2_ film can be mainly indexed to the high‐crystalline‐quality hexagonal molybdenite structure that corresponds to JCPDS card No. 37–1492.

**Figure 2:**
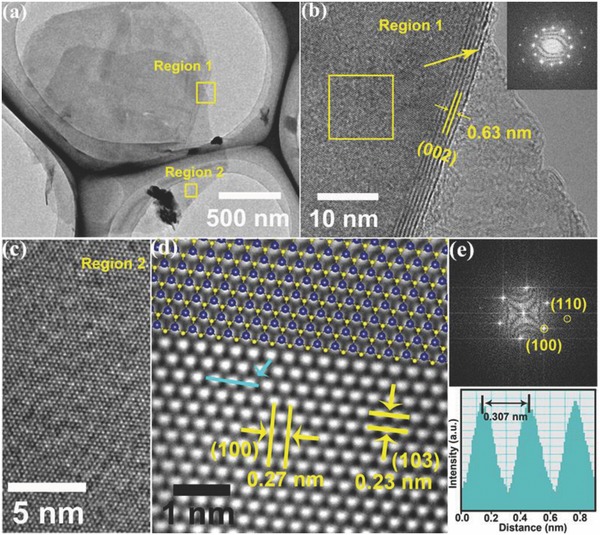
a) TEM image of V‐MoS_2_ nanosheets transferred onto a copper grid, b) HRTEM image of V‐MoS_2_ nanosheets corresponding to region 1 with the inverse FFT image of a selected area in the right‐top corner, c) HRTEM image of V‐MoS_2_ nanosheets corresponding to region 2, d) FFT image with corresponding structure model overlaid, and e) the inverse FFT image (top) and the line intensity profile of the line drawn in the inverse FFT image (bottom).

To further characterize the structure and quality of the MoS_2_ nanosheets, HRTEM images were measured, as shown in **Figure** **2**. The magnified result in Figure 2b (corresponding to the yellow box marked as region 1 in Figure 2a) shows a typical edge view of approximately seven‐layer MoS_2_ nanosheets with clear lattice fringes, which further verifies the Raman and SEM measurement results, and the interlayer spacing is measured to be 0.63 nm, which is comparable with the reported results for few‐layer MoS_2_ film.^38^ The diffraction pattern in the fast Fourier transform (FFT) image of MoS_2_, lying on the left upside layer (yellow square in Figure 2b), is shown in the top right of Figure 2b. Two hexagonal structures are clearly intermixing with each other because of the stack of two exfoliated nanosheets. Then, the yellow box marked as region 2 in Figure 2a was well studied with the HRTEM image shown in Figure 2c. The inverse FFT reconstruction from the FFT pattern is given in the top of Figure 2d. It clearly shows a hexagonally symmetric lattice structure with the MoS_2_ structure model well overlapped, and the *d*‐spacings of the (100) and (103) lattice planes are 0.27 and 0.23 nm, respectively. Figure 2e shows the corresponding FFT reciprocal lattice of Figure 2d, with the (100) and (110) planes labeled; this observation also indicates that the MoS_2_ nanosheet is of hexagonal structure with highly crystalline quality. The distance between the two peaks in the line intensity profile shown in the bottom of Figure 2e is ≈0.307 nm, which matches well with the value of the unit cell parameter of MoS_2_.^39^ 2D materials with high‐quality vertically oriented few‐layer structures have been reported to be ideal materials for photodetectors, energy storage, catalysis, and edge emitter devices because of their high active surface areas, strong light absorptions, and quick longitudinal intralayer transport speeds.^34^, ^35^, ^36^, ^37^, ^40^, ^41^, ^42^ Therefore, these V‐MoS_2_ nanosheets may also have great potential applications in multifarious fields.

## 3 Results and Discussion

**Figure 3:**
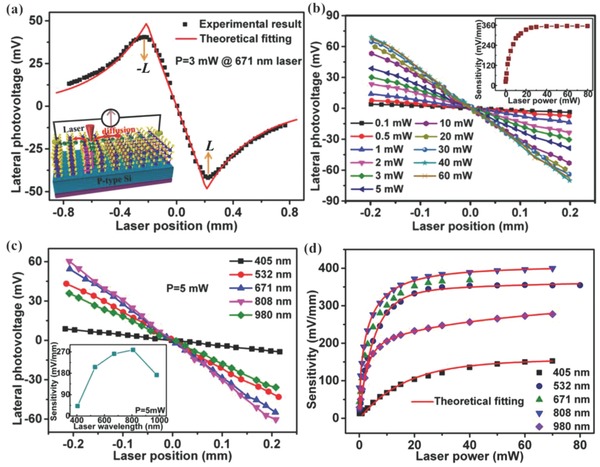
a) A typical LPV as a function of the laser position for the V‐MoS_2_/Si heterojunction with the diagram of the LPE measurement shown in the inset. b) The dependence of the LPV on the laser position under illumination of a 532 nm laser at different laser powers, with in the inset the extracted laser‐power‐dependent position sensitivity. c) The dependence of the LPV on the laser position for different lasers under an illumination of 5 mW, with the extracted laser wavelength‐dependent position sensitivity shown in the inset. d) The position sensitivity as a function of laser power for different lasers.

Here, the V‐MoS_2_ nanosheets were successfully transferred onto a cleaned Si substrate to form the V‐MoS_2_/Si heterojunction, and then well studied by LPE measurement to explore their potential applications in PSDs. For LPE measurement, the sample was scanned spatially with a continuous‐wave semiconductor laser beam (ranging from visible to near‐infrared) focused on an ≈100 µm diameter spot at the MoS_2_ surface, without any spurious illumination reaching the samples. A longitudinal current–voltage (*I*–*V*) curve of the V‐MoS_2_/Si heterojunction on the surface of the MoS_2_ and the back of the Si substrate, which exhibits a good backward diode‐like rectifying behavior at room temperature, suggesting a p–n junction is produced at the interface of the MoS_2_/Si heterojunction with a barrier height of ≈0.45 eV (as shown in Figure S4a, Supporting Information). Before the LPE measurement, two 50 nm thick Au point electrodes were prepared on the MoS_2_ surface by thermal evaporation with a point diameter of 0.5 mm and contact distance of 0.4 mm (controlled by a mask plate) to well form the ohmic contact, as shown in Figure S4b of the Supporting Information. When a light beam is illuminated on the MoS_2_, the light is absorbed by MoS_2_ or Si substrate. Photon‐excited electrons and holes are generated in the junction and then separated into MoS_2_ and Si by the build‐in field. Thus, the electric‐field gradient between the illuminated and the nonilluminated zones results in excess electron diffusion from the illuminated spot toward the electrodes.^43^, ^44^ Therefore, when a light beam is scanned away from electrode A to electrode B on the surface of MoS_2_, an lateral photovoltage (LPV) as a function of light position is observed without adding an extra bias voltage, as shown in **Figure** **3**a. Based on carrier diffusion theory, the LPV can be described as^45^, ^46^ follows

(1) $$\mathrm{LPV}\;=\;K(N_{A}\;-\;N_{B})\;=\;KN_{0}\left[\exp\left(-\frac{\left|x-L\;\right|}{l}\right)\;-\exp\;\left(-\frac{\left|x\;+\;L\;\right|}{l}\right)\right]$$

where *N*_A_ and *N*_B_ are the densities of excess electrons at electrodes A and B, respectively, *K* is the proportionality coefficient, *L* is the half‐distance between the two electrodes, *N*_0_ is the number of the separated electron–hole pairs per second at light position *x*, and *l* is the electron transverse diffusion length in MoS_2_. The LPV curve in Figure 3a can be well fitted by Equation (1), indicating that the LPE can be explained using the carrier diffusion model. Generally, it is supposed that *l* << *L* to obtain the linear relation between LPV and *x* in the region of the two electrodes. Thus, Equation (1) can be simplified as follows^45^, ^46^

(2) $$\mathrm{LPV}\;=\;\frac{2KN_{0}}{l}\exp\;\left(-\frac{L}{l}\right)\;x\;(-L\;\;\leq \;\;x\;\;\leq \;\;L)$$

Clearly, there is a linear relationship between the LPV and the laser spot position when the contact distance of the two electrodes is small, which is the significant characteristic of LPE. Based on this characteristic, the devices may play an important role in the field of PSDs.^44^ From Equation (2), position sensitivity, which is one of the key figures of merit for PSDs, can be written as follows:

(3) $$\mathrm{Position}\;\mathrm{sensitivity}\;=\;\frac{\mathrm{LPV}}{x}\;=\;\frac{2KN_{0}}{l}\exp\;\left(-\frac{L}{l}\right)\;(-L\;\;\leq \;\;x\;\;\leq \;\;L)$$

In reality, the LPV is not exactly linearly proportional to laser position because of the small diffusion length,^46^ inhomogeneous sample layer^47^ or inappropriate thickness,^45^ and asymmetric external field modulation.^48^ To evaluate the distortion of the LPV output, nonlinearity, as another figure of merit for PSDs, is used; a good device is defined as a device having nonlinearity of less than 15%.^49^ From previous reports, the quantity of nonlinearity is defined as follows^44^, ^49^

(4) $$\mathrm{Nonlinearity}=\delta =\frac{2\times \mathrm{RMS}\;\mathrm{deviation}}{\mathrm{Measured}\;\mathrm{full}\;\mathrm{scale}}$$

where RMS is the root mean square deviation from the regression line data.

First, the LPV as a function of laser position was measured in the MoS_2_/Si structure with a contact distance of 0.4 mm under the illumination of a 532 nm laser. The output laser power was modulated from 0.1 to 80 mW through an optical attenuation and measured with a photo power meter (Coherent Field MaxII‐TOP), as shown in Figure 3b. From the LPV curves, we can observe that the linearities are all very good, with maximal nonlinearity of no more than 5.27% at 60 mW based on Equation (4), which suggests that the PSD of this structure has very high measurement accuracy. Moreover, the LPVs are strongly dependent on the laser power. To illustrate this, the position sensitivities are extracted by linear fitting, as shown in the inset of Figure 3b. The position sensitivity increases from 22.7 mV mm^−1^ to a saturated value of 354.5 mV mm^−1^ with increasing laser power from 0.1 to 80 mW, which is an usual phenomenon in metal/semiconductor structures or semiconductor heterostructures due to the competition of increased photogenerated electron–hole pairs and increased carrier recombination rate in the region of irradiation with increasing light intensity.^50^, ^51^, ^52^ Notably, this structure has a very large power response range, and the sensitivity of 22.7 mV mm^−1^ even under such low laser power is much larger than the saturated sensitivity in other systems,^50^, ^53^, ^54^ suggesting that the power responsivity is very high. In addition, the saturated sensitivity of 354.5 mV mm^−1^ largely outshines any previously reported results in other systems,^51^, ^55^, ^56^, ^57^ which may be ascribed to both the strong light absorption (the vertically layered structure allows the incident light to reach the heterojunction interface region easily to ensure the absorbance of as much light as possible) and the quick longitudinal transport of photogenerated carriers in this vertically oriented layered MoS_2_ structure.

To determine the relative sensitivity to different wavelengths, the LPE response was measured under illumination of five different lasers (405, 532, 671, 808, and 980 nm) ranging from visible to near‐infrared, as shown in Figure 3c. The laser power was kept identical (5 mW) for all wavelengths during measurement. It is found that the LPE, along with the extracted position sensitivity (inset of Figure 3c), which first increases with increasing wavelength from 405 to 808 nm, and then tends to decrease at longer wavelengths, exhibits a complex and nonmonotonic dependence on the laser wavelength. Then, we also investigated the relationship of laser wavelength‐dependent LPE under illumination of different laser powers for all lasers, with the extracted position sensitivities, shown in Figure 3d. One can observe that the position sensitivities (/LPEs) highly depend on the laser intensity within the measurement range of laser power and exhibit a laser power dependency similar to that of a 532 nm laser. Moreover, the position sensitivities always maintain a nonmonotonic dependence on the laser wavelength, without depending on the intensity of the laser power, which suggests that the observed laser wavelength‐dependent LPE for these V‐MoS_2_/Si heterojunctions is an intrinsic property, not a random phenomenon.

By Equations (2) and (3), the laser power‐ and wavelength‐dependent LPEs in this V‐MoS_2_/Si structure can be well explained. Equation (2) suggests that the LPE is strongly related to *N*_0_ and *K* under a constant contact distance. When the laser illuminates the MoS_2_ film surface, the laser energy is largely absorbed by the MoS_2_ layer or Si substrate to generate electron–hole pairs; thus, *N*_0_ should be strongly dependent on the number of photons, with the relation $N_{0}\;=\;\kappa {\left(\frac{P\lambda }{hc}\right)}^{\alpha }$ (where *P* is the laser power, *h* is the Planck constant, λ is the laser wavelength, *c* is the speed of light in vacuum, and κ and α are the proportionality coefficients with 0 < κ, α < 1). Moreover, *K* is mainly related to the carrier lifetime and the laser‐power‐dependent carrier recombination rate, which can be expressed as $K=1-\delta^{\beta \tau P/N_{0}}$ (where δ is the recombination rate, β is a proportionality coefficient, and τ is the carrier lifetime). Substituting *K* and *N*_0_ into Equation (3) yields^52^, ^58^ the following equation

(5) $$\begin{array}{l} \mathrm{Position}\;\mathrm{sensitivity}=\frac{2\kappa {\left(P\lambda \right)}^{\alpha }}{l{\left(hc\right)}^{\alpha }}\exp\left(-\frac{L}{l}\right)\\ \;\;\;\;\;\;\;\;\;\;\;\;\;\;\;\;\;\;\;\;\;\;\;\;\;\;\;\;\;\times \;(1\;-\;\delta^{\beta \tau \;{\left(hc\right)}^{\alpha }P^{1-\alpha }/\lambda^{\alpha }\kappa })(-L\;\;\leq \;\;x\;\;\leq \;\;L) \end{array}$$

It seems that the position sensitivity should be proportional to the laser wavelength without considering other influence factors. Considering this contradiction with our experimental results, it is suggested that the photo quantum efficiency (proportional to κ) should play an important role in this structure, as is usually reported in other photovoltaic or optoelectric devices. When we measured the external quantum efficiency (EQE), it was observed that this structure has a very large light response broadband, ranging from 350 to 1100 nm (as shown in Figure S5, Supporting Information). More importantly, the EQE tendency is consistent with the spectral response of the LPE in the inset of Figure 3c, indicating that the wavelength‐dependent position sensitivity is mainly determined by the photo quantum efficiency of the laser wavelength. In addition, we fitted the laser‐power‐dependent position sensitivities for different lasers using Equation (5). As indicated by the red solid lines in Figure 3d, the theoretical fittings are in good accordance with our experimental results with the best‐fitting parameters of 0.02 < α < 0.095, 0.46 < δ < 0.91, 0.65 × 10^−3^ < β < 1.9 × 10^−3^, 1.3 × 10^−3^ < κ < 3.4 × 10^−3^, and τ ≈ 1.21 × 10^−5^, which further indicates that the laser‐power‐dependent LPEs are indeed induced by the coexistence of increased photogenerated electron–hole pairs and increased recombination rate. More importantly, it is worth pointing out that the saturated position sensitivity reaches a supramaximal value of 401.1 mV mm^−1^ at 808 nm and 277.3 mV mm^−1^ at 980 nm, suggesting that the V‐MoS_2_/Si heterojunction is of great importance for the application of LPE in broadband PSDs, especially in the infrared range.

**Figure 4:**
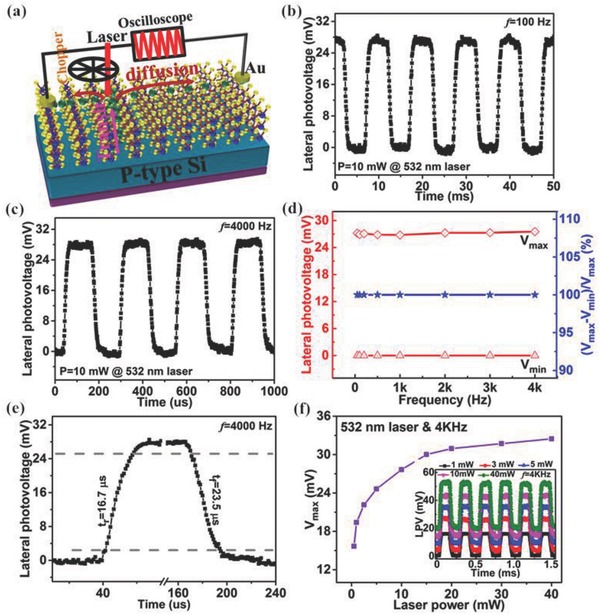
a) The schematic illustration of the setup for studying the time response of V‐MoS_2_/Si heterojunction PSD. Time‐dependent lateral photovoltages at frequencies of b) 50 Hz and c) 4000 Hz. d) *V*_max_, *V*_min_, and relative balance (*V*_max_ − *V*_min_)/*V*_max_ versus chopper frequency. e) The magnified plots of one response cycle at a frequency of 4000 Hz. f) Laser‐power‐dependent *V*_max_, with the time‐dependent lateral photovoltages under illumination of five typical laser powers at a frequency of 4000 Hz shown in the inset.

Then, the response speed, which is another key figure of merit for an optical photodetector,^30^, ^33^, ^49^ is investigated in detail in the V‐MoS_2_/Si heterojunction. Generally, the response speed of a PSD should be higher than the fastest temporal variation in the signal and should have a frequency response that has a bandwidth covering the entire bandwidth of the signal to get a faithful optical signal. To evaluate the response speed of this MoS_2_/Si heterojunction, a pulsed lateral photovoltage signal was measured first by combining a laser chopper and a digital oscilloscope, as in the schematic diagram shown in **Figure** **4**a. During the measurement, a pulsed laser, which was obtained by tuning a continuous‐wave laser through a chopper with frequency ranging from 10 to 4000 Hz, was used to illuminate the MoS_2_/Si heterojunction at a constant laser spot position (*x* = 0.15 mm). Figure 4b,c shows the time‐dependent lateral photovoltage responses at typical frequencies of 100 and 4000 Hz, respectively, under illumination of a constant laser power (10 mW). It was found that both the *V*_max_ (laser on) and the *V*_min_ (laser off) nearly do not decrease or increase and the relative balance (*V*_max_ – *V*_min_)/*V*_max_ is always 100% over the whole frequency range (shown in Figure 4d), which suggests that the V‐MoS_2_/Si heterojunction has a very wide frequency response range. From the magnified lateral photovoltage curve at 4000 Hz in Figure 4e, a rise time of 16.7 µs and a fall time of 23.5 µs can be obtained, respectively. Moreover, we also measured the time‐dependent lateral photovoltages under different laser powers (averaged) ranging from 1 to 40 mW, with the typical results at 4000 Hz shown in the inset of Figure 4f. It is observed that the response time is independent of the laser power. In addition, the lateral photovoltage balance values remain nearly constant for each laser power without depending on the chopper frequency, and *V*_max_ increases gradually until becoming saturated with increasing laser power, as shown in Figure 4f, which is consistent with the LPE results. More importantly, it is noteworthy that the response time of this heterojunction is much faster than those of most reported MoS_2_‐based photodetectors.^10^, ^11^, ^12^, ^30^, ^59^, ^60^, ^61^, ^62^, ^63^ Though ultrafast and stable photoresponse with no degradation, as well as resistance capacitance (RC)‐limited bandwidth, of the optoelectric devices is one of the basic requirements for applications in high‐speed optical communications, until now, the frequency of the modulated laser has been lower than 4000 Hz in most of the previously reported nano‐PDs, which will hinder their application in dynamic real‐time detection of the optical signal. However, the rise (/fall) time of this V‐MoS_2_/Si heterojunction at 4000 Hz obviously cannot represent its true response speed, as the relative balance of lateral photovoltage ((*V*_max_ – *V*_min_)/*V*_max_) does not decrease in the measurement frequency range, and the resolution of the response time, to some extent, is related to the laser‐pulse frequency and width.

**Figure 5:**
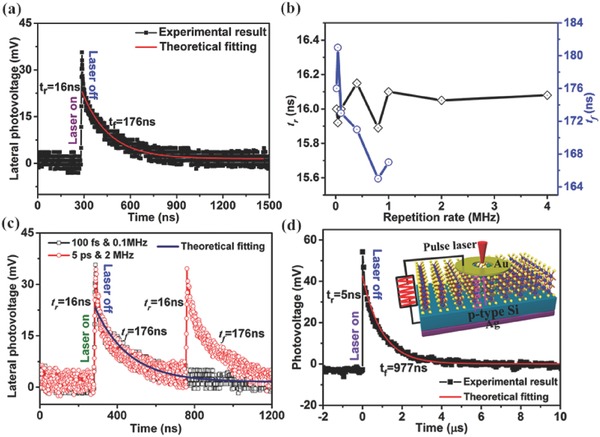
a) The time‐dependent lateral photovoltage for one pulse illumination (pulse width of 100 fs), b) *t*_r_ and *t*_f_ as functions of the repetition rate. c) The time‐dependent lateral photovoltages under illumination of pulsed lasers with pulse widths of 100 fs and 5 ps. d) The time‐dependent photovoltage of the V‐MoS_2_/Si heterojunction photodetector for one pulse illumination (pulse width of 100 fs) with inset the schematic illustration of the setup.

Considering the saturation characteristic of the laser‐power‐dependent lateral photovoltage and the pulse frequency and width‐dependent resolution of the response time, it was suggested that a pulsed laser with very short pulse width may be used to obtain the intrinsic response time. Then, we further investigated the response speed of the V‐MoS_2_/Si heterojunction using an oscilloscope to monitor the variation of the lateral photovoltage under the illumination of a pulsed light at the same position. The pulsed light (800 nm) was produced from a 100 fs‐pulse‐width Ti:sapphire laser (Spectra‐Physics, Mai Tai HP) using a pulse selector to modulate the laser repetition frequency ranging from 16 KHz to 4 MHz. The typical measurement result at a frequency of 0.1 MHz and laser power of 10 mW (averaged) is shown in **Figure** **5**a. The peak photovoltage is nearly the same as the saturated value in the inset of Figure 4f. More importantly, the rise (/relaxation) time reaches an amazing value of 16 ns (/176 ns), and the response speed is nearly independent of the laser repetition rate (the time range should be enough large to ensure the accuracy of the fitted parameters, so that the relaxation times are not given for frequencies larger than 1 MHz), which implies that the pulse width may be a very key factor in determining the response time, as shown in Figure 5b. Then, to determine the potential relation between the pulse width and the response time, we also measured the response speed of the MoS_2_/Si heterojunction under the illumination of another pulsed light with a pulse width of ≈5 ps and a repetition rate ranging from 2 to 78 MHz (NKT SuperK EXTREME), as shown in Figure 5c. Interestingly, the response speed is very consistent with that of the 100 fs‐pulse‐width laser. Comparing the response times with the ultrashort pulse widths, it is suggested that the rise time and fall time should be independent of the pulse width, provided the pulse width is much shorter than the rise time. From the above time‐response measurements, it is clear that the extracted response speed is nearly independent of both the repetition rate and the pulse width under the illumination of ultrashort pulse lasers, and this V‐MoS_2_/Si heterojunction PSD is capable of working at the very high frequency of 4 MHz. However, to obtain the accurate response speed, the repetition rate cannot be too large, especially relative to the relaxation time (meaning that the pulse interval should be enough long to ensure the recovery of the lateral photovoltage).

To verify the influence of the pulse width on the response speed, we designed an experiment to measure the response speed of LPE in the V‐MoS_2_/Si heterojunction by tuning the pulse width. (The measurement diagram is shown in Figure S6a, Supporting Information.) The output laser of 532 nm with a beam diameter of ≈1 mm was focused with a lens (focal length = 100 mm) before reaching the sample. The chopper was placed at three different positions (position 1: outside the lens; position 2: between the lens and the sample, and close to the lens; position 3: between the lens and the sample, and close to the sample), thus the pulse widths were different because of the different beam diameters under the same chopper frequency. The lateral photovoltage responses were measured at the three different chopper positions with a frequency of 4000 Hz (as shown in Figure S6b, Supporting Information). It is clear that the response time gradually decreases from 16.7 (/23.5) µs to 6.9 (/11.2) µs with decreasing pulse width. When the laser is turned on or off, the laser illumination needs to maintain the pulse width so that the lateral photovoltage does not get the balance value during this period, which indicates that the measured response speed is strongly dependent on the laser pulse width when the pulse width is longer than the rise (/fall) time; this may be why the response time decreases clearly with decreasing laser pulse width. However, when the pulse width is much shorter than the rise (/fall) time, the lateral photovoltage response starts to proceed nearly after one pulse illumination; thus, the measured response speed should be independent of the laser pulse width and should be intrinsic. Therefore, the same response time measured under illumination of fs‐ or ps‐pulse‐width pulsed laser represents the real response speed of the V‐MoS_2_/Si heterojunction PSD.

From the working principle of PSD shown in Figure 4a, it is clear that the carrier transport in LPE contains both a longitudinal and a transverse process, which is very different from that of the photodetector. Therefore, the response time of PSD may be longer than that of the photodetector as transverse carrier transport is also needed in LPE for PSD based on longitudinal transport. Moreover, though it is usually suggested that the intralayer transport speed of photogenerated carriers is much larger than the interlayer speed,^33^, ^34^, ^41^ the quantitative difference between them is still unknown experimentally because of the single longitudinal transport process in the MoS_2_‐based photodetector, from which only the interlayer (parallel‐oriented layered structure) or intralayer (vertically oriented layered structure) transport time can be obtained. However, using the response speed of the PSD may be a good method to extract both the interlayer and the intralayer transport speeds simultaneously. To obtain both the longitudinal and the transverse response times, we also measured the longitudinal response speed of the photogenerated carriers in the V‐MoS_2_/Si heterojunction with a photodetector; the schematic diagram is shown in the inset of Figure 5d. The single‐pulse photovoltage curve at 100 fs pulse width and 0.1 MHz repetition frequency in Figure 5d shows that the rise and the relaxation times are estimated to be 5 and 977 ns, respectively. By combining with the above results of the PSD, the transverse layer‐to‐layer transport time should be ≈11 ns. Obviously, the intralayer transport speed is much faster (≈2.2 times) than the interlayer speed, and, to the best of our knowledge, this may be the first experimental verification. The high response speed of the V‐MoS_2_/Si heterojunction as a PSD can be mainly ascribed to the following three probable reasons. The first is that the quality of the CVD‐prepared MoS_2_ is very high with relatively fewer defects/traps, and the Si substrate is extensively cleaned with a new thin SiO_2_ passivation layer on it; thus, the defect recombination in the bulk and interface can be largely reduced as these defects is suggested to have significantly influence on the photoelectric properties.^26^ The second is that the distinct vertically standing few‐layer structure of the MoS_2_ film can greatly facilitate the longitudinal transport of photogenerated carriers along the intralayer direction to the top collection electrode, resulting in high response speed and large LPV. However, the measured response time is still less than the transmit time (<1 ns) of carriers from the junction interface to the top electrode by assuming a mobility of 200 cm^2^ V^−1^ s^−1^ 2, 64 and a layer thickness of 2 µm for the MoS_2_ layer, which can be attributed to both the incompletely vertically oriented layered structure of MoS_2_ and the relatively slow transmit time of carriers from the junction interface to the bottom electrode for the Si substrate. The third is that the strong built‐in electric field at the MoS_2_ and the Si interface can further enhance the separation and transport of photogenerated carriers. Moreover, for both the PSD and photodetector, when the laser is turned off, the separated carriers in the top layer will tunnel back by longitudinal in‐plane transport to recombine with the carriers in the bottom layer, thus their relaxation times should be the same. Considering the inconsistency with our results, it is suggested that this can be ascribed to the higher number of recombination channels in the PSD than in the photodetector, as the separated carriers can only tunnel back to the bottom layer through the intralayer transport around the laser spot position in the photodetector but can tunnel back to the bottom layer through the intralayer transport in the whole measurement range in the PSD, as shown in Figures 4a and 5d.

At last, we also investigated the air stability of the V‐MoS_2_/Si PSD by placing it directly in air for two months without any encapsulation. Both the LPE response and the speed response remain unchanged, respectively (as shown in Figure S7a,b, Supporting Information), which demonstrate that the device is stable for practical application in the PSD. From the above results, it is clear that this large‐area, high‐quality, and uniformly continuous V‐MoS_2_/Si heterojunction PSD exhibits very high and stable photoresponse performance, which is the basis for achieving the commercialization of high‐performance photodetectors based on the V‐MoS_2_/Si heterojunction.

## 4 Conclusion

In conclusion, the large‐area, high‐quality, and vertically oriented few‐layer MoS_2_ nanosheets were prepared and successfully transferred onto the Si substrate to form the V‐MoS_2_/Si heterojunction. LPE measurements suggest that the V‐MoS_2_/Si heterojunction PSD exhibits excellent linearity and ultrahigh position sensitivity in the visible‐near‐infrared range without the application of an external bias voltage. Moreover, an ultrafast response speed of 16 ns was found in the PSD by time‐response measurements, which is the best result achieved for MoS_2_‐based photodetectors to date. More importantly, the V‐MoS_2_/Si heterojunction PSD exhibits excellent stability and reproducibility for both the lateral photovoltaic and the time responses. The performance of this PSD is significantly better compared to that of the PSDs of other MoS_2_ or Si‐based heterojunctions. The high LPE performances and ultrafast response time can be attributed to the good quality of the V‐MoS_2_/Si heterojunction with strong light absorption and quick carrier transport speed in the unique vertically oriented few‐layer MoS_2_ nanosheets and the large built‐in electric field at the interface of V‐MoS_2_ and Si. Furthermore, by combining these results with the response speed measurement in the V‐MoS_2_/Si heterojunction photodetector, the intralayer transport time of 5 ns and the interlayer transport time of 11 ns were extracted experimentally. Our results suggest that the CVD‐prepared large‐area, high‐quality, and vertically oriented few‐layer MoS_2_/Si heterojunction shows great potential for applications in high‐performance optoelectronic devices.

## 5 Experimental Section

*MoS_2_ Film Deposition*: MoS_2_ nanosheets with a vertically standing layered structure were synthesized on the Si/SiO_2_ substrate by the CVD technique, according to the diagram shown in Figure S1a of the Supporting Information. MoO_3_ powders and sulfur pellets were used as the raw materials. The growth temperature profiles are shown in Figure S1b of the Supporting Information. First, the sulfur and the MoO_3_ heating zones were heated to 65 and 160 °C, respectively, and the temperatures were maintained for 40 and 30 min, respectively, to remove the vapor from the raw material and system. Then, the heating zones were heated to 150 and 760 °C for MoS_2_ preparation. During the process, Ar was added into the quartz tube as a carrier gas with a flow rate of 35 sccm under the atmospheric pressure. After ≈20 min, the large‐area, high‐quality, and V‐MoS_2_ nanosheets were prepared with height of 2 µm.

*Characterizations of V‐MoS_2_ Nanosheets*: The structure and morphology of the as‐prepared MoS_2_ films were identified by XRD (Bruker D8 Advance), HRTEM (FEI, Tecnai G2), SEM (FEINova NanoSE M450), and EDXS (EDAX OCTANE PLUS). The optical properties were measured using Raman spectroscopy (Horiba JobinYvon, LabRAM HR Evolution). The chemical states were obtained using XPS (Thermo Fisher, ESCALAB250Xi).

*MoS_2_/Si Heterojunction Preparation*: The MoS_2_ nanosheets on an Si/SiO_2_ substrate were first spin‐coated with polymethyl methacrylate (PMMA); then the substrate was placed on a hot plate, heated for 30 min at 80 °C and immersed in an NaOH solution (2 mol L^−1^) at 60 °C until the PMMA thin film could be removed from the substrate; the film was transferred to deionized water overnight to wash away the residual NaOH, lifted out with a prepared Si substrate and baked at 80 °C for 2 h to remove the water residue and eliminate the possible wrinkles; acetone was used to dissolve the organic PMMA. Afterward, low‐temperature annealing (≈200 °C) was carried out in vacuum chamber to strengthen the adhesion of V‐MoS_2_ to the Si substrate. Finally, the Au (50 nm) top point electrode and Ag (100 nm) bottom electrode were prepared on the surface of MoS_2_ and the back of Si substrate, respectively, by thermal evaporation.

*LPE and Time‐Response Measurements*: The current–voltage (*I*–*V*) curves were measured with a Keithley 2400 SourceMeter. The LPV was measured using a Keithley 2700 voltmeter and a 3D electric motorized stage with a continuous‐wave laser of different wavelengths (405, 532, 671, 808, and 980 nm) as the illumination source. The response speed of the V‐MoS_2_/Si heterojunction was evaluated by combining a light chopper (or fs‐ and ps‐pulse‐width pulsed lasers) and a digital oscilloscope (Agilent DSO X 4022A).

## Conflict of Interest

The authors declare no conflict of interest.

## Supporting information

Supplementary
