# Supplementary material for: Ultrahigh, Ultrafast, and Self‐Powered Visible‐Near‐Infrared Optical Position‐Sensitive Detector Based on a CVD‐Prepared Vertically Standing Few‐Layer MoS2/Si Heterojunction
Source: Adv Sci (Weinh). 2017 Dec 1;5(2):1700502. doi: 10.1002/advs.201700502 (PMC5827457; doi:10.1002/advs.201700502)
Supplement: Supplementary file 1 — Supplementary [file ADVS-5-1700502-s001.pdf]

Copyright WILEY-VCH Verlag GmbH & Co. KGaA, 69469 Weinheim, Germany, 2017.

ADVANCED SCIENCE
Open Access

Supporting Information

for *Adv. Sci.,* DOI: 10.1002/advs.201700502

Ultrahigh, Ultrafast, and Self-Powered Visible-Near-Infrared Optical Position-Sensitive Detector Based on a CVD-Prepared Vertically Standing Few-Layer MoS_2/Si Heterojunction

Ridong Cong, Shuang Qiao, Jihong Liu, Jiansong Mi, Wei Yu, Baolai Liang, Guangsheng Fu, Caofeng Pan,* and Shufang Wang**

Supporting Information

Ultrahigh, ultrafast, and self-powered visible-near-infrared optical position-sensitive detector based on a CVD-prepared vertically standing few-layer MoS_2/Si heterojunction

Ridong Cong, Shuang Qiao, Jihong Liu, Jiansong Mi, Wei Yu, Baolai Liang, Guangsheng Fu, Caofeng Pan*, and Shufang Wang**

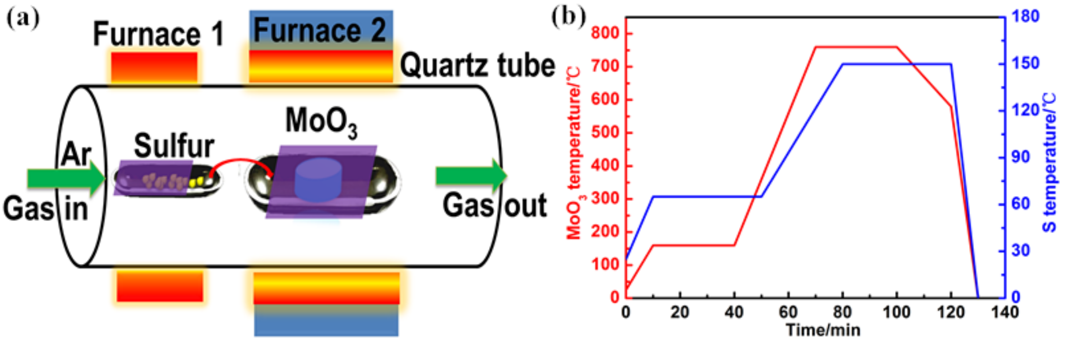

Figure S1. a) Schematic diagram of MoS_2 growth, and b) Growth temperature profile.

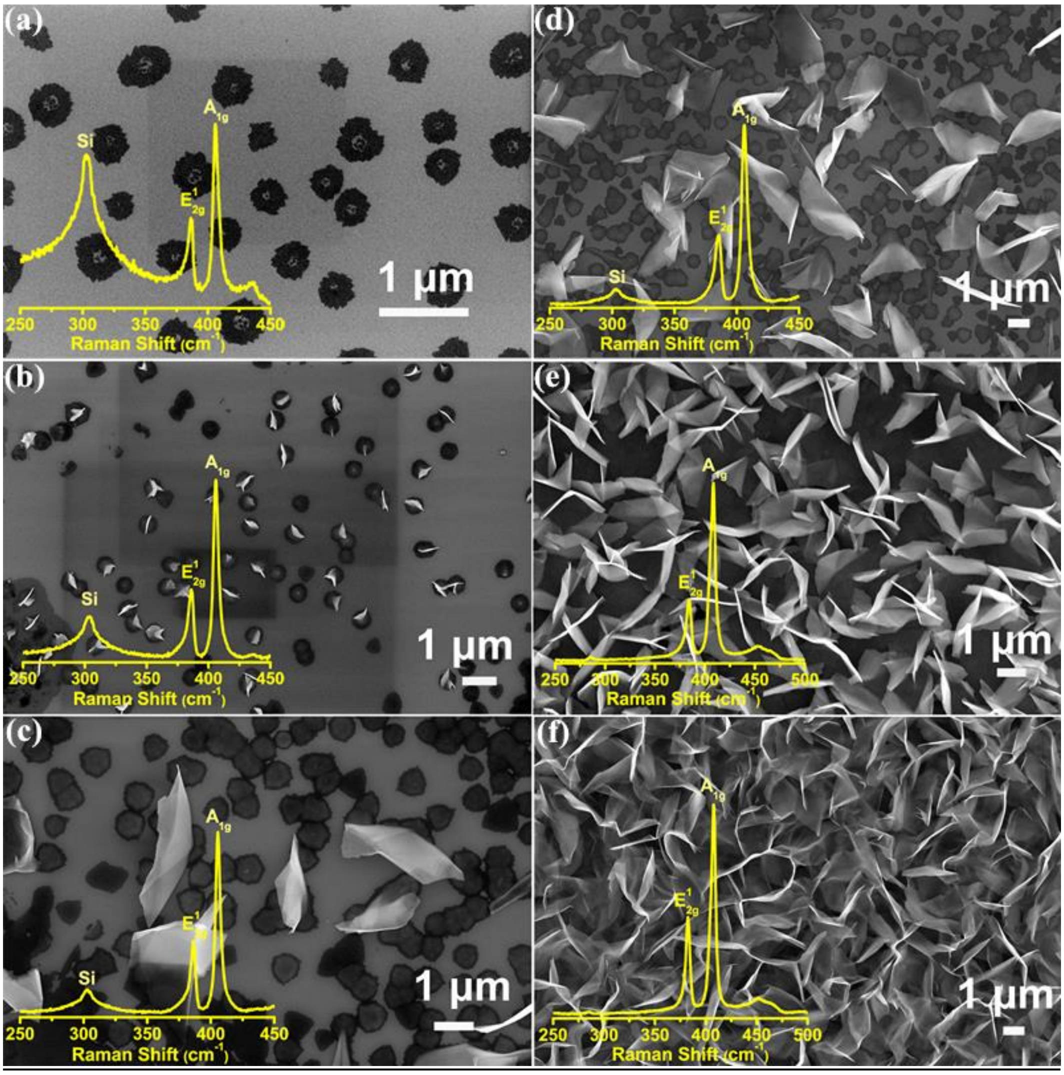

Figure S2. SEM images of V-MoS_2 nanosheets synthesized on SiO_2/Si substrate with growth times of a) 2 min, b) 4 min, c) 6 min, d) 10 min, e) 15 min, and f) 20 min. The insets in the bottom-left corners are the corresponding Raman spectra.

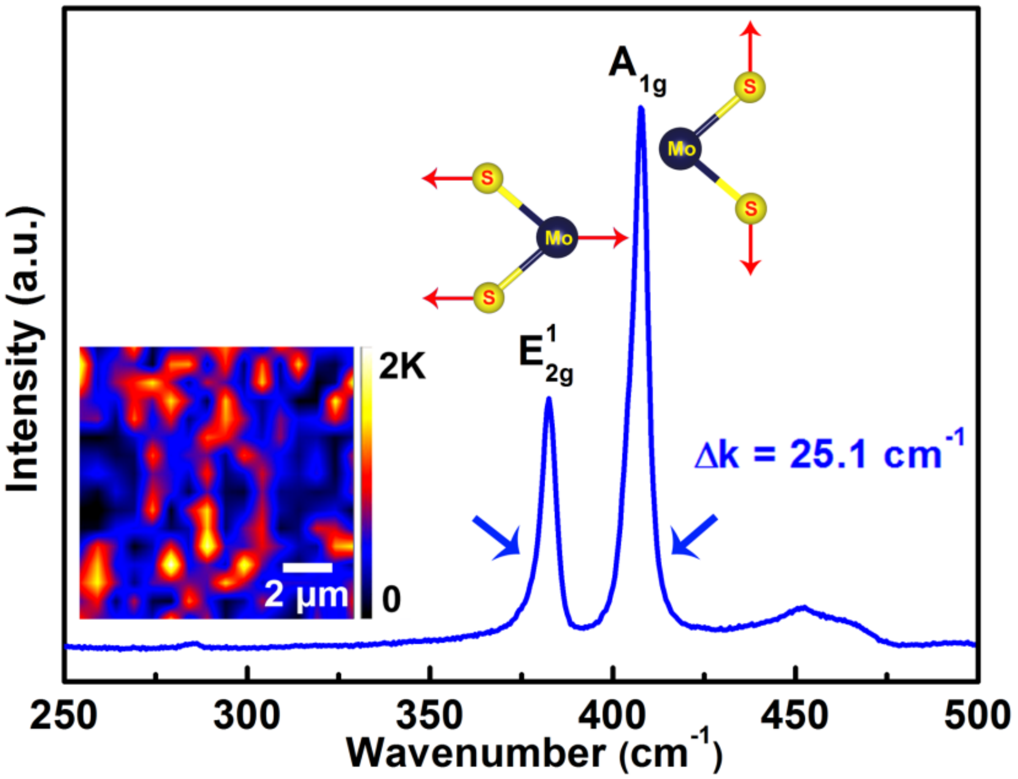

Figure S3. Raman spectrum of the prepared V-MoS_2 nanosheets with the Raman mapping result in the inset.

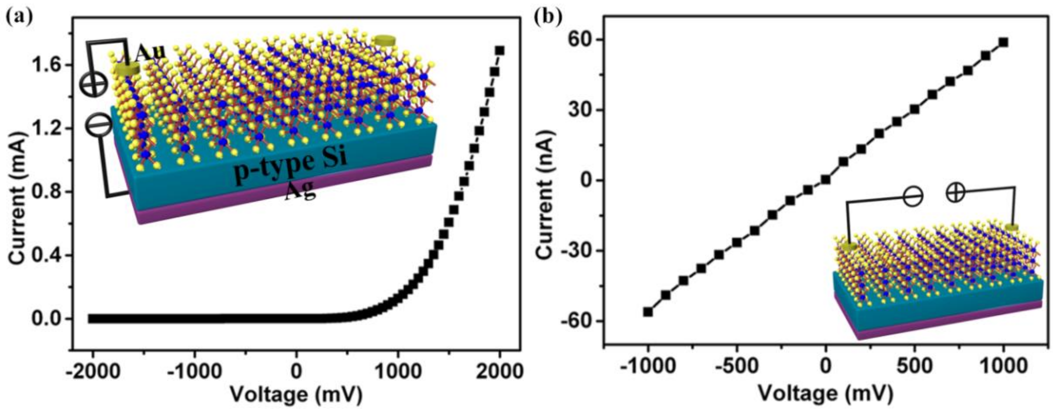

Figure S4. a) Longitudinal, and b) Transverse I-V curves for the V-MoS_2/Si heterojunction with the corresponding diagrams of I-V measurements shown as insets.

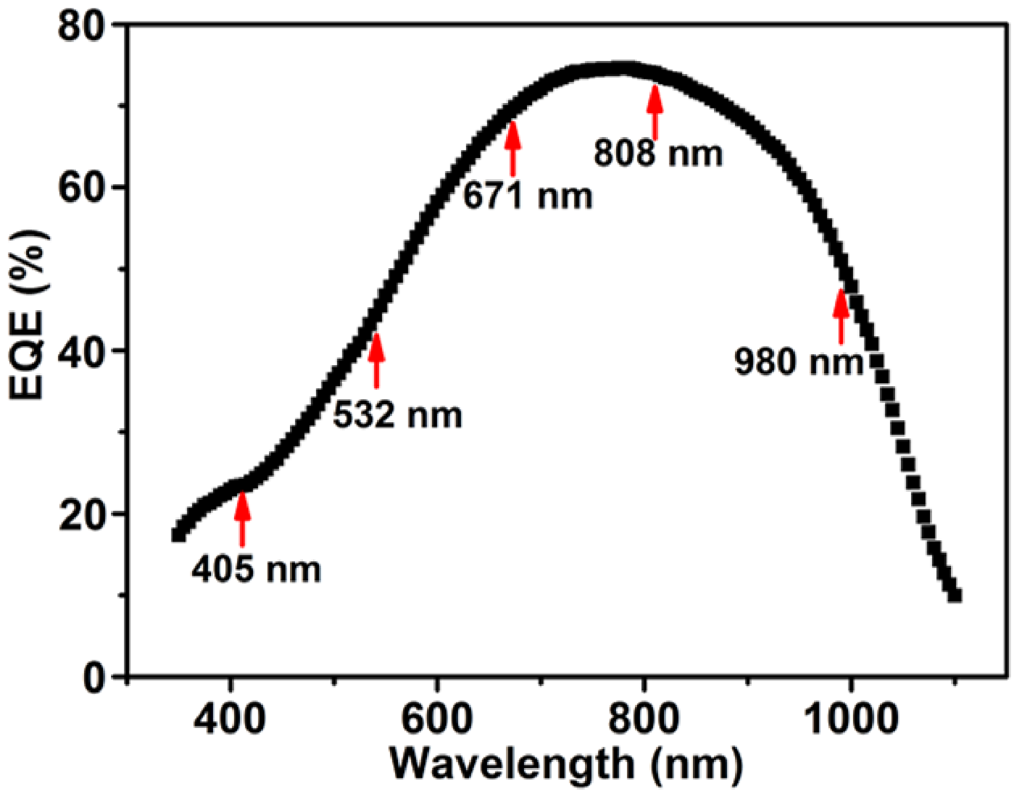

Figure S5. EQE spectrum of the V-MoS_2/Si heterojunction.

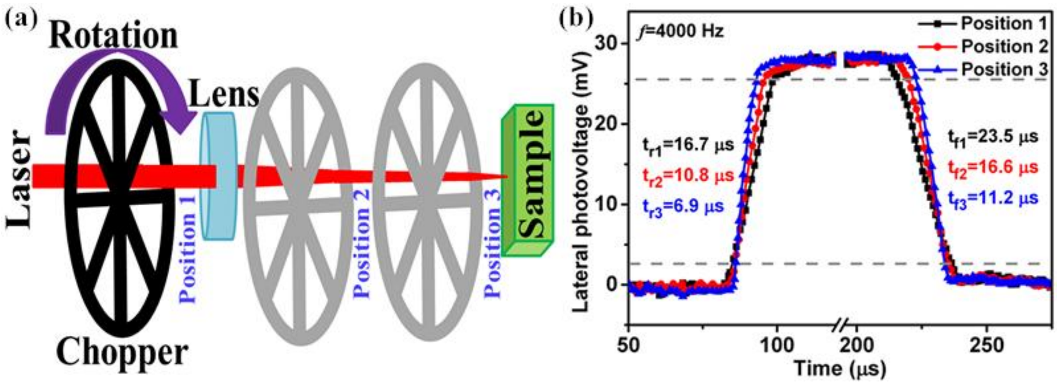

Figure S6. a) The diagram of the time response with the chopper at three different positions for obtaining different pulse widths, b) The magnified plots of one response cycle under different chopper positions at a frequency of 4000 Hz.

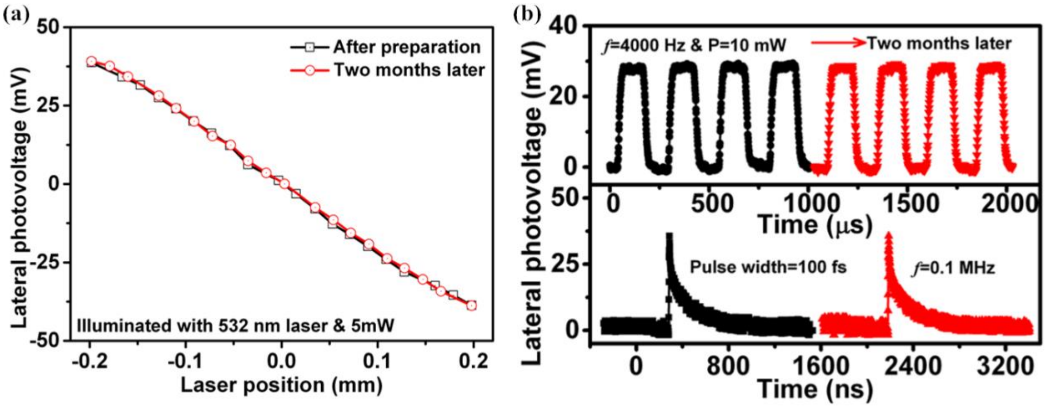

Figure S7. a) The dependence of the LPVs on the laser position, and b) Time-dependent lateral photovoltage response (Top: chopper-induced pulse laser, Bottom: 100 fs-pulse-width pulsed laser) of the as-fabricated device and the same device after storage in air for two months.
